# Evaluation of a new live recombinant vaccine against cutaneous leishmaniasis in BALB/c mice

**DOI:** 10.1186/s13071-020-04289-7

**Published:** 2020-08-12

**Authors:** Samira Salari, Iraj Sharifi, Ali Reza Keyhani, Pooya Ghasemi Nejad Almani

**Affiliations:** 1 Leishmaniasis Research Center, Kerman University of Medical Sciences, Kerman, Iran; 2 Students Research Committee, Kerman University of Medical Sciences, Kerman, Iran; 3 Medical Mycology and Bacteriology Research Center, Kerman University of Medical Sciences, Kerman, Iran; 4 Department of Medical Parasitology and Mycology, Kerman University of Medical Sciences, Kerman, Iran

**Keywords:** Live recombinant vaccine, *L. tarentolae*, KMP11 antigen, LACK antigen

## Abstract

**Background:**

Leishmaniasis is a serious health problem in some parts of the world. In spite of the many known leishmaniasis control measures, the disease has continued to increase in endemic areas, and no effective vaccine has been discovered.

**Methods:**

In this study, *Leishmania tarentulae* was used as a living factory for the production of two LACK and KMP11 immunogenic antigens in the mice body, and safety profiles were investigated. The sequences of the KMP11 and LACK *L. major* antigens were synthesized in the pLEXSY-neo 2.1 plasmid and cloned into *E. coli* strain Top10, and after being linearized with the *SwaI* enzyme, they were transfected into the genome of *L. tarentolae*. The *L. tarentolae-LACK/KMP11/EGFP* in the stationary phase with CpG ODN as an adjuvant was used for vaccination in BALB/c mice. Vaccination was performed into the left footpad. Three weeks later, the booster was injected in the same manner. To examine the effectiveness of the injected vaccine, pathogenic *L. major* (MRHO/IR/75/ER) was injected into the right footpad of all mice three weeks following the booster vaccination. In order to assess humoral immunity, the levels of IgG1, and IgG2a antibodies before and 6 weeks after the challenge were studied in the groups. In addition, in order to investigate cellular immunity in the groups, the study measured IFN-γ, IL-5, TNF-α, IL-6 and IL-17 cytokines before, 3 weeks and 8 weeks after the challenge, and also the parasite load in the lymph node with real-time PCR.

**Results:**

The lowest level of the parasitic load was observed in the G1 group (mice vaccinated with *L. tarentolae-LACK/KMP11/EGFP* with CpG) in comparison with other groups (*L. tarentolae*-LACK/KMP11/EGFP +non-CpG (G2); *L. tarentolae*-EGFP + CpG (G3, control); *L. tarentolae*-EGFP + non-CpG (G4, control); and mice injected with PBS (G5, control). Moreover, the evaluation of immune response showed a delayed-type hypersensitivity towards Th1.

**Conclusions:**

According to the results of this study, the live recombinant vaccine of *L. tarentolae-LACK/KMP11/EGFP* with the CpG adjuvant reduced the parasitic load and footpad induration in infected mice. The long-term effects of this vaccine can be evaluated in volunteers as a clinical trial in future planning.
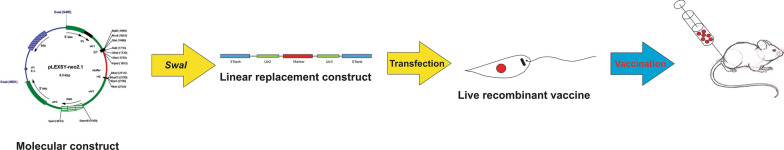

## Background

According to the WHO report [1], leishmaniasis is a serious health problem in some parts of the world, especially in the Eastern Mediterranean region. There are four forms of this disease in these regions, zoonotic and anthroponotic visceral leishmaniasis (ZVL/AVL), and zoonotic/anthroponotic cutaneous leishmaniasis (ZCL/ACL), with the highest infection rates belonging to the cutaneous leishmaniasis based on the number of reported cases [2–5]. ZVL, ACL and ZVL types are present in 14 out of 22 countries in this region including Afghanistan, Egypt, Iraq, Iran, Jordan, Libya, Pakistan, Morocco, Saudi Arabia, Yemen, Somalia, Sudan, Syria, and Tunisia. Each type can exist in multiple foci within endemic regions. The cases of infection have been increasing over the past 10 years in the aforementioned countries [6]. In 2018, 100,000 new cases of this disease were reported to the WHO from these countries. The cases reported from Iran consisted of 8649 cases of ACL and 18175 cases of ZCL [7–9].

The control of leishmaniasis, especially of its zoonotic types, has not been successful in many countries. No human vaccine has yet been reported for this disease, and the treatment of the cutaneous type is only based on pentavalent antimonial compounds. Due to side effects and increased drug resistance [10, 11], further studies are required to discover effective medication and vaccines for this disease.

The *Leishmania* homologue of the receptor for the activated C kinase (LACK) antigen is highly conserved in *Leishmania* species, expressed on both promastigotes and amastigotes [12]. In a recent study, the injection of the newly recombinant LACK antigen against *L. major* stimulated CD8^+^ and increased interferon γ (IFN-γ) in the lymph nodes of mice and conferred protective immunity to mice infected with *L. major* [13]. In patients with CL lesions, the use of the LACK antigen produced IFN-γ and IL-10 in patients with localized cutaneous leishmaniasis during the early stages of infection [14]. In peripheral blood mononuclear cells exposed to the parasite, the LACK antigen increased T CD8^+^ and NK cells [15].

The Kinetoplastida membrane protein-11 (KMP11) antigen is another immunogen antigen in *Leishmania* spp., which is expressed in both amastigote and promastigote stages. This protein was first introduced by Jardim et al. [16] as a T-cell interacting protein in *Leishmania* spp., with a strong antigenicity to stimulate mouse and human T-cells and capable of stimulating both innate and acquired immune systems.

KMP11 can stimulate both inherent and acquired immune systems with minimum homology with human proteins, making it a good candidate for use in the production of vaccines [16]. The use of KMP11 and hydrophilic acylated surface protein B (HASPB) antigens as vaccine DNA on *L. major* in BALB/c mice increases IgGa2 and IFN-γ in vaccinated mice and reduces the parasitic load in lymph nodes and the spleen [17].

*Leishmania tarentolae* is a protozoan that is non-pathogenic for humans, receiving attention in recent years due to its role as the host for the production of recombinant proteins. The unique qualities of this host include the presence of a glycosylation pattern similar to that of mammals, easy and cost-effective culture, high homogeneity of the glycosylated protein, and high expression. Due to these properties of *L. tarentolae*, this host has replaced prokaryotic hosts [18].

*Leishmania tarentolae* can survive in BALB/c mice, and its injection to mice effectively targets the dendritic cells and lymphoid organs, thereby increasing antigen presentation and the level and quality of T-cell immune responses. It has been applied as a vaccine against *Leishmania donovani*, inducing Th1, and increasing immunity in mice [19]. In the present study, *L. tarentolae* was employed as a live factory producing two effective antigens inside the body of mice, and the immune profiles were studied.

## Methods

### Production of recombinant parasites

*Leishmania tarentolae* Tar II strain (ATCC 30.267) was cultured at 26 °C and pH 7.2 in BHI medium enriched with thermally inactivated 20% fetal bovine serum (FBS), 5 µg/ml hemin, and Pen-Strep containing 10,000 IU of penicillin and 10,000 μg of streptomycin (base)/ml (Jena Bioscience, Jena, Germany). EGFP and LACK-KMP11-EGFP genes were synthesized inside a pLEXSY-neo 2.1 vector (Jenna Bioscience, Jena, Germany) by Bioneer (Daejeon, South Korea). PLEXSY-neo 2.1/LACK-KMP11-EGFP and pLEXSY-neo2.1/EGFP vectors were cloned inside the *Escherichia coli* strain Top10. For the transfection of the pLEXSY-neo2.1/LACK-KMP11-EGFP pLEXSY-neo2.1/EGFP construct inside the genome of *L. tarentolae*, the construct was linearized using the *Swa*I enzyme (New England Biolabs, Ipswich, MA, USA). For the transfection of the linearized constructs inside the parasite, the physical method with electrical voltage was adopted using the Xcell GENE PULSER electroporation device (Bio-Rad, Hercules, USA) with CE and PC modulators based on the manual of Jenna Bioscience. In two sterile 1.5 ml microtubes, 350 µl of the sediment containing the parasite in the BHI medium (in which 1 × 10^8^ parasites existed in each ml) was taken and poured in every microtube. Then, 10 µg of the linearized pLEXSY-neo2.1/ EGFP and PLEXSY-neo2.1/ LACK-KMP11-EGFP constructs were added to the microtubes and pipetted, and these mixtures were added to two 400 µl cuvettes (d = 2 mm) of the device between the two electrodes.

These cuvettes were incubated on ice for 10 min and placed separately in the electroporation device and pulsated at 450 V and 450 Fµ. Subsequently, the transfectants were transferred to the BHI medium containing Pen-Strep, Hemin, and 20% FBS and incubated for 24 h at 26 °C. After this period, the neomycin antibiotic (LEXSY Neo; Jenna Bioscience) (1000× stock solution, 50 mg/ml) was added to the culture medium for the drug selection of transfectants. To confirm the integration of constructs inside the genome of the parasite in the *SSU* locus on the DNA extracted from the transfectants, PCR was performed with P1442 (5′-CCG ACT GCA ACA AGG TGT AG-3′) as the forward primer and A264 (5′-CAT CTA TAG AGA AGT ACA CGT AAA AG-3′) as the reverse primer.

To examine the expression of GFP genes, the *L. tarentolae*-LACK/KMP11/EGFP and the *L. tarentolae*- EGFP promastigotes were investigated under a fluorescence microscope (Nikon Eclipse E400, Japan). They were centrifuged at 3000× rpm for 10 min and were re-suspended after washing in PBS once and placed on the microscope slide. To check the production of LACK/KMP11 proteins in *L. tarentolae*-LACK/KMP11/EGFP, the proteins secreted from the BHI culture medium containing the *L. tarentolae* parasite were extracted following the method based on Jena Bioscience company instructions. Ten ml of the culture medium containing the parasite was centrifuged at 3000×*g* at 4 °C. The supernatant was filtered using a 30 mm syringe filter unit 0.22 µm (Jet Biofil™, Guangzhou, China). Then, 2 ml of cooled 50% trichloroacetic acid (TCA) was added to 8 ml of the filtered supernatant. This solution was kept on ice for 30 min and then centrifuged at 15,000×*g* at 4 °C for 15 min. The supernatant was disposed of, and the sediment was dissolved in 1 ml of 80% acetone. This solution was centrifuged at 15,000×*g* at 4 °C for 15 min. The supernatant was removed, and 80 µl gel loading buffer was added to the sediment. After 5 min of boiling, 10 µl of the proteins were separated by SDS PAGE on 12% polyacrylamide gel using the vertical electrophoresis tank (Mini PROTEAN Tetra Cell; Bio-Rad, USA). Protein bands were transferred to the PVDF membrane (Thermo Fisher Scientific, Waltham MA, USA) by the semi-dry blotting device (Bio-Rad, Hercules, USA)

In the next step, a blocking solution (TBS with 3% skimmed milk) was poured over the membrane and placed in a refrigerator for 1 h. Then, the Anti-His-tag antibody (Abcam, USA, ab14923) was added to the membrane as the primary antibody to detect the LACK protein, as a His-tag was placed in the molecular structure at the end of this protein. Also, the Anti S-tag antibody (Abcam, Cambridge, MA, USA, ab183674), as the primary antibody, was added to the membrane to detect the KMP11 protein, considering that an S-tag had been placed in the molecular structure at the end of this protein. Next, the Goat anti-rabbit IgG alkaline phosphatase conjugate, the secondary antibody, (Abcam, Cambridge, MA, USA, ab6722) was added to the membrane. After incubation and washing, alkaline phosphatase buffer and the NBT/BCIP substrate solution (Sigma-Aldrich, St. Louis, USA) were added and incubated in the dark at room temperature (22–25 °C) to view the bands.

### Preparation of antigens

The antigens *L. tarentolae*-LACK/KMP11/EGFP, *L. tarentolae*-EGFP, and *L. major* (MRHO/IR/75/ER) were required for assessing the response of antibodies. After culturing these parasites in the RPMI 1640 medium and reaching the stationary phase, they were washed in triplicate with the PBS (1.75 mM KH_2_PO_4_, 8 mM Na_2_HPO_4_, 137 mM NaCl, and 0.25 mM KCl) buffer. The antigen was produced using the freeze-thaw (F/T) method with 10 times exposure to liquid nitrogen and then placed in a 37 °C water bath [20]. The Pierce BCA Protein Assay Kit (Thermo Fisher Scientific) was used to measure the antigen concentration.

### Vaccination

The inbred female BALB/c mice aged 6–8 weeks and weighing 20–25 g were purchased from the animal house of Pasteur Institute of Iran. They were kept under standard conditions and fed with water and rodent pellet diet, 12:12 light:dark cycle, and 50% humidity. Mice were divided into 5 groups, each consisting of 20 mice. The parasite in the stationary phase was used for vaccination. Vaccination was performed in the left footpad (Table 1). Three weeks later, the booster was injected in the same manner. The vaccinated groups included the following:

**Table 1:** Groups of vaccinated mice in the present study

Mice group	Prime	Boost	Challenge	Modality
G1	*L. tarentolae*-LACK/KMP11/EGFP adjuvant CpG	*L. tarentolae*-LACK/KMP11/EGFP adjuvant CpG	*L. major*	rLive vaccine
G2	*L. tarentolae*-LACK/KMP11/EGFP adjuvant nCpG	*L. tarentolae*-LACK/KMP11/EGFP adjuvant non-CpG	*L. major*	rLive vaccine
G3	*L. tarentolae*-EGFP adjuvant CpG	*L. tarentolae*-EGFP adjuvant CpG	*L. major*	Control
G4	*L. tarentolae*-EGFP adjuvant nCpG	*L. tarentolae*-EGFP adjuvant non-CpG	*L. major*	Control
G5	PBS	PBS	*L. major*	Control

Group 1: vaccination was performed with 2 × 10^7^ *L. tarentolae*-LACK/KMP11/EGFP parasites plus 20 µg of CpG (5-TCCATGACGTTCCTGACGTT-3) (InvivoGen, San Diego, USA) as the adjuvant. Group 2: vaccination was performed with 2 × 10^7^ *L. tarentolae*-LACK/KMP11/EGFP parasites plus 20 µg of non-CpG (5-TCCAGGACTTCTCTCAGGTT-3) (InvivoGen) as the control ODN without CpG motifs. Group 3: vaccination was performed with 2 × 10^7^ *L. tarentolae*-EGFP parasites plus 20 µg of CpG (5-TCCATGACGTTCCTGACGTT-3) (InvivoGen) (control group). Group 4: vaccination was performed with 2 × 10^7^ *L. tarentolae*-EGFP parasites plus 20 µg of non-CpG (5-TCCAGGACTTCTCTCAGGTT-3) (InvivoGen) (control group). Group 5: PBS was injected in this group (control group). To examine the effectiveness of the injected vaccine, pathogenic *L. major* of a well-defined strain (MRHO/IR/75/ER) in 2 × 10^5^ cells/50 µl was injected in the right footpad of all mice three weeks after the booster vaccination.

### Antigen-specific antibody response

To examine the antibody response before the injection of the *L. major* parasite, and six weeks after the injection of this parasite, blood samples were taken from the tail of the mice from all groups. To examine the humoral immune response, the IgG1 and IgG2a antibodies were measured using ELISA method, and the IgG2a/IgG1 ratio against the (10 µg/ml) *L. tarentolae*-LACK/KMP11/EGFP F/T (10 µg/ml) *L. tarentolae*- EGFP (10 µg/ml) *L. major* antigens were also calculated. The antigen in the coating buffer (Carbonate-bicarbonate buffer, pH 9.6) was placed in the wells of ELISA microplates (Greiner Bio-One, Frickenhausen, Germany) and kept overnight at 4 °C. After that, the plates were washed four times in PBS + 0.05% Tween 20. Next, 200 µl of the blocking buffer (BSA 1% in PBS) was added to each well and kept at 37 °C for 2 h. After washing four times, 100 µl of the 1:100 dilutions of each serum sample of the mice groups was added to each well. Then, the plates were kept at 37 °C for 2 h. After washing four times, 100 µl of the antibodies conjugated with HRP goat anti-mouse (IgG1-HPR) (R&D System, Minneapolis, USA) prepared in the antibody diluting solution (1:10,000 dilution) (for the IgG1 assay) or goat anti-mouse IgG2a HPR (R&D system) (1:10,000 dilution) (for the IgG2a assay) was added to each well. The plates were placed in the incubator at 37 °C for 2 h. After washing four times, 100 µl of the ABTS substrate solution (Thermo Fisher Scientific) was added to each well. The plates were incubated at 37 °C for 30 min. Then, 100 µl of the stop solution (SDS 1%) was added to each well, and the plates were read at the wavelength of 405 nm using an ELISA Reader (BioTek, USA). The tests were performed in triplicate for all groups.

### Cytokine assays

To examine cellular immunity, IL-6, TNF-α, IL-5, IFN-γ and IL-17 cytokines were examined before the injection of the stationary phase of *L. major* and then 3 and 8 weeks after the injection of the *L. major*. In each group, three mice before the injection of pathogenic *L. major* and 3 and 8 weeks after the injection of *L. major* (MRHO/IR/75/ER) were sacrificed in adherence to the standards of a painless death, the spleens were separated, and their cells were homogenized in the homogenizer device. To eliminate red blood cells (RBCs), the red blood cell ACK Lysing Buffer (0.15 M NH_4_Cl, 0.1 mM Na2EDTA, and 1 mM KHCO_3_) (Thermo Fisher Scientific) was added for 5 min. Next, spleen cells were washed using the full DMEM medium (DMEM medium including 5% FBS, 0.1% L glutamine, 1% HEPES, 0.1 2ME, and 0.1% gentamicin), and a homogeneous solution of the spleen cells were obtained. After counting the cells with a Neubauer slide, 3.5 × 10^6^ cells were added to the wells of a 24-well plate for culturing (Jet Biofil, Guangzhou, China). *L. tarentolae*-LACK/KMP11/EGFP F/T (20 µg/ml) (before challenge), *L. tarentolae*- EGFP F/T (20 µg/ml) (before challenge), and *L. major* F/T antigens (20 µg/ml) (3 and eight 8 after challenge) were added to the wells. The well without any antigen and only containing the culture medium and cells were considered as the negative control, and the well containing Concanavalin A at 5 mg/ml concentration and cells was regarded as the positive control. The plates were incubated at 37 °C and 5% CO_2_. Measurement of IL-6, TNF-α, IL-5, IL-17 and INF-ɣ cytokines from the supernatant of the cell culture after 24, 72 and 96 h, respectively, using a kit (sandwich ELISA; R&D System Kits, USA) according to the manufacturer’s guide. The tests were performed in triplicate for all groups.

### Measurement of footpad induration, ulceration and parasite load with real-time PCR

The footpad induration and ulceration of the mice were measured and recorded every week up to 11 weeks after the injection with *L. major* (MRHO/IR/75/ER) parasite using vernier calipers. Real-time PCR was performed to measure the parasite load in the mice. At weeks 8 and 11 after the injection of *L. major* (MRHO/IR/75/ER), three mice were randomly selected from each group, euthanized under standard conditions without pain, and were dissected. The popliteal lymph node and the footpad were separated and weighed, and then used to extract DNA using the DNA extraction kit (Gene All, Seoul, South Korea). Real-time PCR reaction was performed using the Rotor-Gene Q (Qiagen, Germany) using RV1 (forward: 5′-CTT TTC TGG TCC CGC GGG TAG G-3′) and RV2 (reverse: 5′-CCA CCT GGC CTA TTT TAC ACC A-3′) primers [21]. In this method, by plotting the standard curve, the parasite load in injected mice was calculated after DNA extraction in comparison to the standard curve. To plot the standard curve, consecutive dilutions of genomic DNA of *L. major* at the final concentration of 1–10^5^ parasites were prepared. In each reaction, at least one parasite could be detected in the test. All tests were performed in triplicate. Each reaction consisted of 30 ng of 5 pmol DNA of each forward and reverse primer, and 5 µl of Qiagen QuantiFast SYBR Green Master Mix at the final volume of 12 µl. PCR reaction conditions were 95 °C for 5 min, 25 cycles as 94 °C for 15 s, 60 °C for 20 s, 60 °C for 20 s, and 72 °C for 20 s.

### Statistical analyses

Charting and analyzing data were done with the Graph-Pad Prism 0.8 software by Tukey *post-hoc* test, ANOVA, and Mann-Whitney U-test. *P-*value of < 0.05 was considered significant.

## Results

### Production of recombinant parasites

Recombinant parasites *L. tarentolae*-LACK/KMP11/EGFP and *L. tarentolae*-EGFP expressing antigens LACK/KMP11/EGFP and EGFP were constructed by transfecting their linear structure inside the locus of *18S* rRNA, *L. tarentolae*. To confirm the stability of recombinant parasites in the expression of transfected proteins, after 30 times passage of the parasites in the culture medium, PCR and Western blot were performed.

**Fig. 1:**
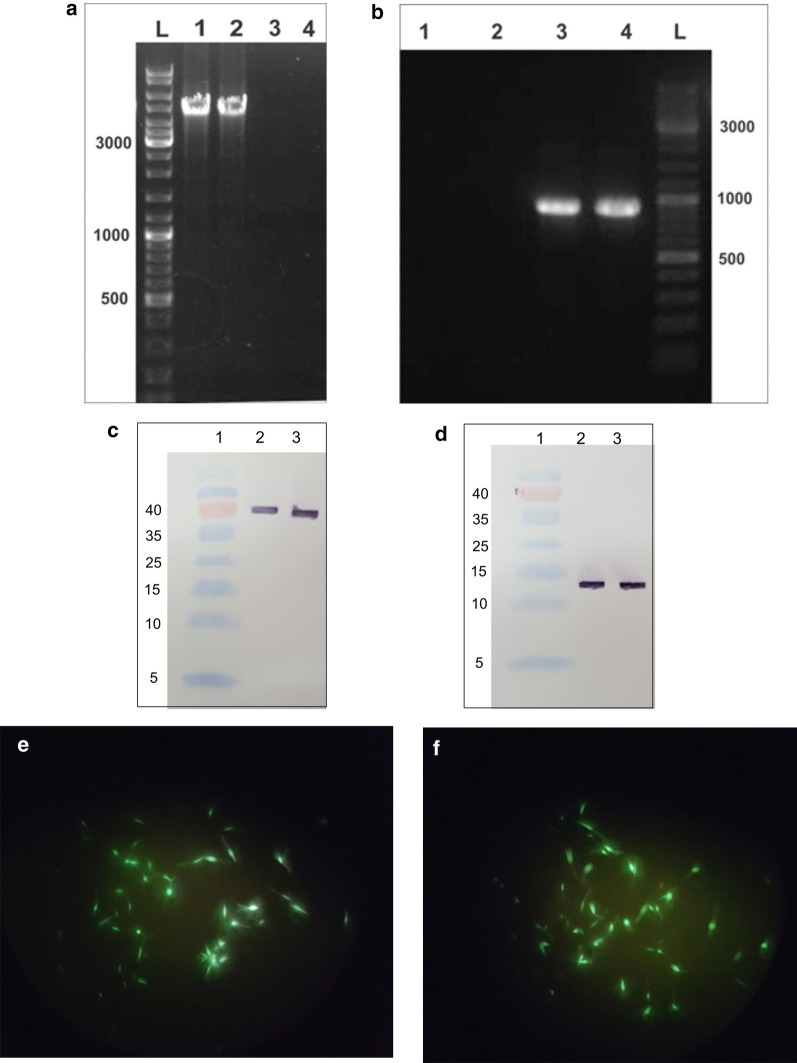
Production of recombinant parasites. **a** Confirmation of integrating pLEXSY-neo2.1/LACK-KMP11-EGFP and pLEXSY-neo2.1/EGFP into the parasite genome by PCR. PCR was performed on the genome of transfectants that show the 4295-bp band (Lanes 1, 2) representative of this gene in the *L. tarentolae-LACK/KMP11/EGFP*. PCR was performed on the genome of the wild parasite, which does not show a band (negative control) (Lanes 3, 4). **b** PCR was carried out on the genome of *L. tarentolae*- EGFP, which shows the 918-bp band representative of this gene in the *L. tarentolae*- EGFP strain (Lanes 3, 4). PCR was carried out on the genome of the wild parasite, which does not show a band (control) (Lanes 1, 2). **c** Confirmation of the LACK protein by Western blot analysis. Lanes 2, 3 produced 38 kDa bands from the LACK protein extracted from the culture medium of recombinant *L. tarentolae*-LACK/KMP11/EGFP parasite using Anti-Hig-tag antibody. **d** Western blot analysis conducted to confirm KMP11 protein. Lanes 2, 3 produced 13 kDa bands from the KMP11 protein extracted from the culture medium of recombinant *L. tarentolae*-LACK/KMP11/EGFP parasite using Anti-S-tag antibody. **e** The recombinant live vaccine of *L. tarentolae*-LACK/KMP11/EGFP containing pLEXSY-neo2.1/LACK-KMP11-EGFP constructs observed by fluorescent microscopy and (**f**) *L. tarentolae*-EGFP promastigotes

PCR was performed to confirm the presence of these genes inside the genome of transgenic *L. tarentolae*. Amplification of the 918-bp sequence for EGFP (Fig. 1b) and 4295-bp sequence for the LACK/KMP11/EGFP gene confirmed their presence (see Fig. 1a). The expression of secreted proteins of LACK and KMP11 in the culture medium was examined using Western blot, and, in the stationary phase, in the *L. tarentolae*-LACK/KMP11/EGFP culture medium. For the detection of LACK protein considering the placement of His-tag at the end of this protein in the construct, the Anti-Hig-tag antibody was used, with the 38 kDa band appearing (Fig. 1c). For the detection of KMP11 protein, due to the placement of S-tag at the end of this protein in the construct, the Anti-S-tag antibody was used, with the 13 kDa band emerging (Fig. 1d). The EGFP expression analysis in the *L. tarentolae*-LACK/KMP11/EGFP (Fig. 1e) and *L. tarentolae*- EGFP was verified using a fluorescent microscope (Fig. 1f). After molecular confirmatory tests and confirmation of the ability of recombinant parasites to produce antigens, the live recombinant vaccine was evaluated.

### Antigen-specific antibody response

**Fig. 2:**
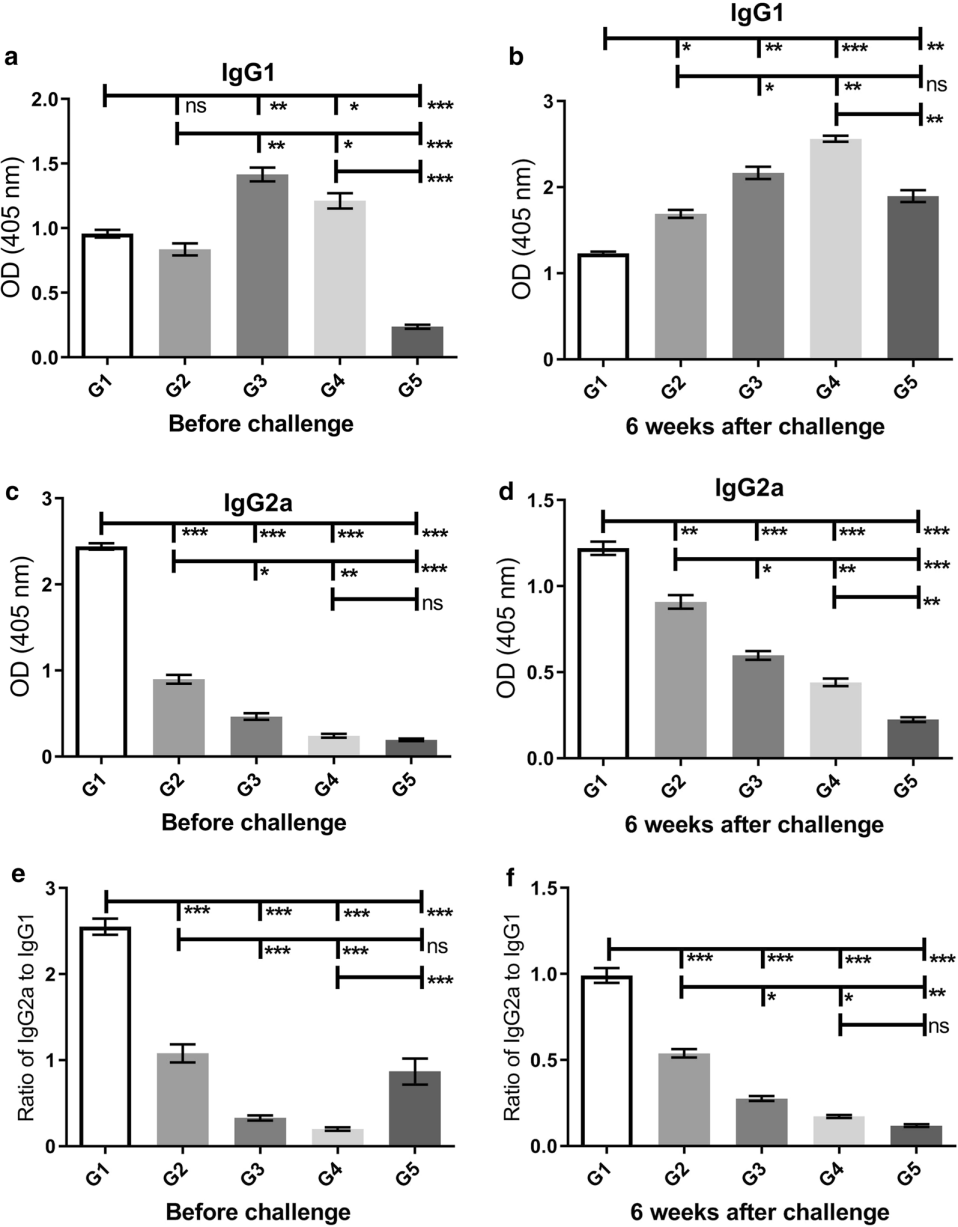
Analysis of the specific humoral responses before and six weeks after challenge. Before injection of the wild parasite (*L. major* (MRHO/IR/75/ER)), sera from all groups were evaluated for anti-*L. tarentolae*-LACK/KMP11/EGFP F/T antigen using ELISA. At 6 weeks after the challenge, *L. major* F/T antigen was tested for all groups. **a**, **b** Assessment of IgG1 levels in sera collected from groups of mice. **c**, **d** IgG2a. All antibody ODs represent as Mean ± SD in each bar. **e**, **f** The ratio of IgG2a/IgG1. Statistical analysis of the data was performed with Mann-Whitney U-test (*P < 0.05, **P < 0.001, ***P < 0.0001; ns, non-significant)

The levels of IgG1 and IgG2a antibodies in all groups were measured before and six weeks after the challenge using ELISA. Moreover, the IgG2a/IgG1 ratio was analyzed in all groups. The production of IgG2a before the challenge against the *L. tarentolae*-LACK/KMP11/EGFP antigen was at a significantly higher level (Mann-Whitney test: *U* = 0, *P *< 0.0001) in the G1 group in comparison to other groups (G2–G5) (Fig. 2c). At this time point, the higher level (Mann-Whitney test: *U* = 0, *P *< 0.001) of IgG1 production was observed in the G3 group in comparison to other groups (Fig. 2a). Six weeks after the challenge, in response to the *L. major* F/T antigen, the highest level of IgG2a was observed in the G1 group and the lowest level was seen in the G5 group (Mann-Whitney test: *U* = 0, *P *< 0.0001) (Fig. 2d). At the same time, the G1 group had a significantly lower level of IgG1 in comparison to other groups (G2–G5) (Mann-Whitney test: *U* = 0, *P *< 0.001) (Fig. 2b). According to Fig. 2e, f, the highest level of IgG2a/IgG1 ratio were observed in the G1 group which was vaccinated with the recombinant *L. tarentolae*-LACK/KMP11/EGFP along with the CpG adjuvant both before and six weeks after the challenge (Mann-Whitney test: U = 0, *P *< 0.0001). This indicates a shift in immunity towards Th1, which was also confirmed by cellular immunity findings.

### Cytokine assays

To examine cellular immunity of IL-6, TNF-α, IL-5, IFN-γ and IL-17 cytokines, before the challenge and 3 and 8 weeks after injection, the supernatant of spleen cells collected from vaccinated and control mice groups in response to F/T. *L. tarentolae-LACK/KMP11/EGFP*, *L. tarentolae/EGFP* (before the challenge) and *L. major* F/T (3 and eight 8 after challenge) were examined.

**Fig. 3:**
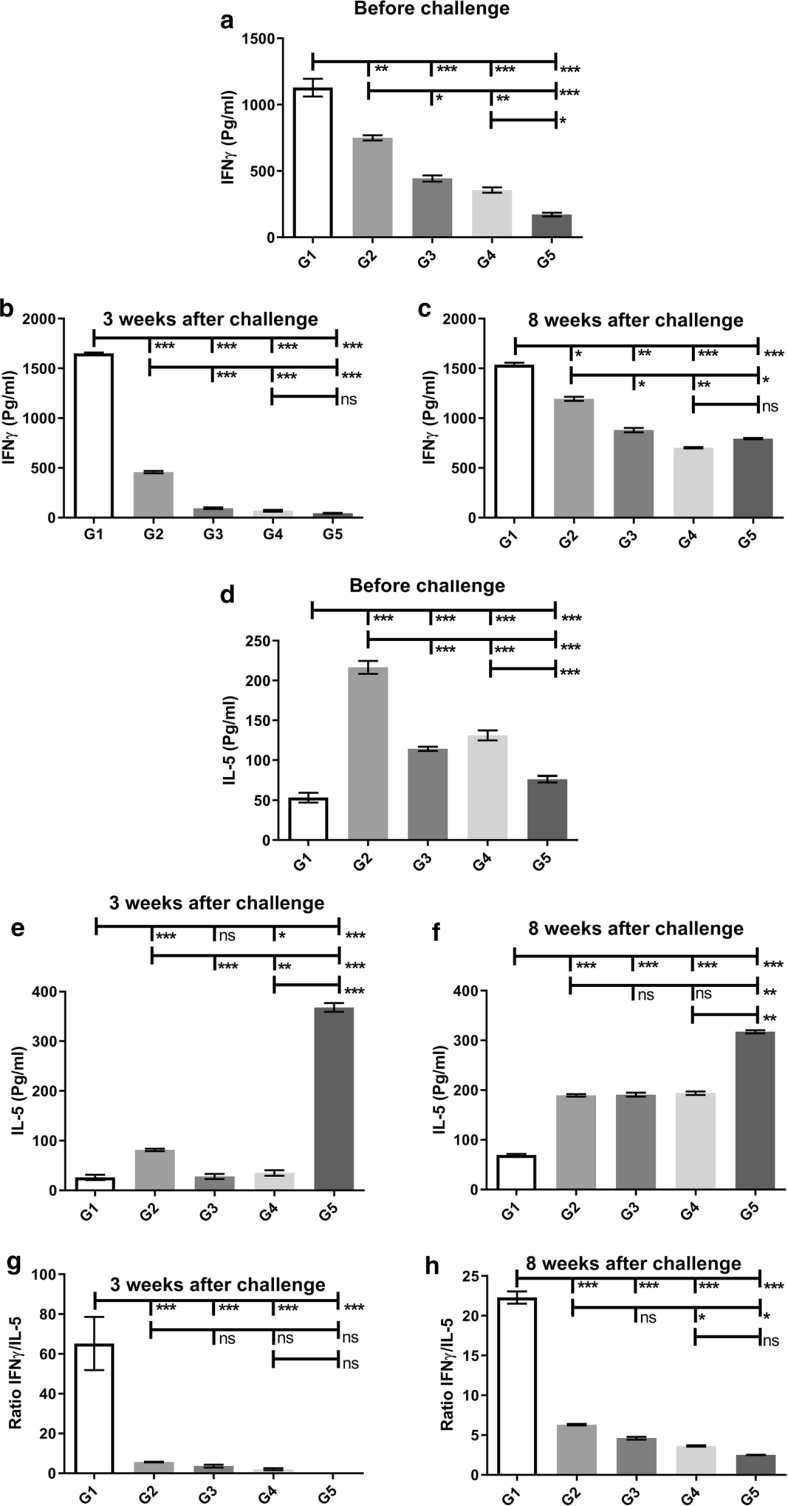
Analysis of the cellular immunity of vaccinated and control BALB/c mice before, 3 weeks, and 8 weeks after last vaccination. IFN-γ (**a**–**c**), IL-5 (**d**–**f**), IFN-γ /IL-5 ratio cytokines (**g**, **h**) after stimulation with *L. tarentolae*-LACK/KMP11/EGFP, *L. tarentolae/EGFP* (before challenge) and *L. major* F/T antigens (3 and eight 8 after challenge) were evaluated by ELISA. All cytokines are represented as the mean ± SD in pg/ml in each bar. Statistical analysis of the data was performed with Mann-Whitney U-test (*P < 0.05, **P < 0.001, ***P < 0.0001; ns, non-significant)

IFN-γ showed the highest level before and after the challenge in the G1 group compared to other groups (Mann-Whitney test: *U* = 0, *P *< 0.0001) (Fig. 3a). Eight weeks after the challenge, the production of IFN-γ in response to the *L. major* F/T antigen was at the highest level in both groups G1 and G2 in comparison to other groups (G3–G5) (Mann-Whitney test: *U* = 0, *P *< 0.0001) (Fig. 3c). For further examination, IL-5 cytokine-dependent on Th2 response was also measured in all groups. Before the challenge, the G1 group had the lowest level of IL-5 production against the *L. tarentolae*-LACK/KMP11/EGFP F/T antigen in comparison to other groups (G2–G5) (Fig. 3d). At the same time, the G2 group had the highest level of IL-5 production against the *L. tarentolae*-LACK/KMP11/EGFP F/T antigen in comparison to other groups (Fig. 3d). Furthermore, 3 and 8 weeks after the challenge, G1 group showed the lowest level of IL-5 production against the *L. major* F/T antigen compared to other groups (G2–G5) (Mann-Whitney test: *U* = 0, *P *< 0.0001) (Fig. 3e, f). On week 3 after the challenge, the G2 group also showed a decrease in the level of IL-5 production than before challenge, but this decrease was greater in G1 group (Mann-Whitney test: *U* = 0, *P *< 0.0001) (Fig. 3e). On the 8th week 8 after the challenge, no significant difference in terms of IL-5 was seen in groups G2, G3 and G4 (Fig. 3f). The G5 group had significantly the highest level of IL-5 (Mann-Whitney test: *U* = 0, *P *< 0.0001) 3 and 8 weeks after the challenge in comparison to other groups (G1–G4) (Fig. 3e, f), indicating a shift in cellular immunity towards Th2.

The IFN-γ/IL-5 ratio was also calculated after the injection of the wild parasite. The G1 group had significantly the highest level of IFN-γ/IL-5 ratio 3 and 8 weeks after injection of *L. major* in comparison to other groups (G2–G5) (Mann-Whitney test: *U* = 0, *P *< 0.0001) (Fig. 3g, h), indicating a shift in cellular immunity towards Th1. The G2 Group also showed the highest level of IFN-γ/IL-5 ratio 3 and 8 weeks after injection of *L. major* after G1 group compared to control groups (Mann-Whitney test: *U* = 0, *P *< 0.0001) (Fig. 3g, h).

**Fig. 4:**
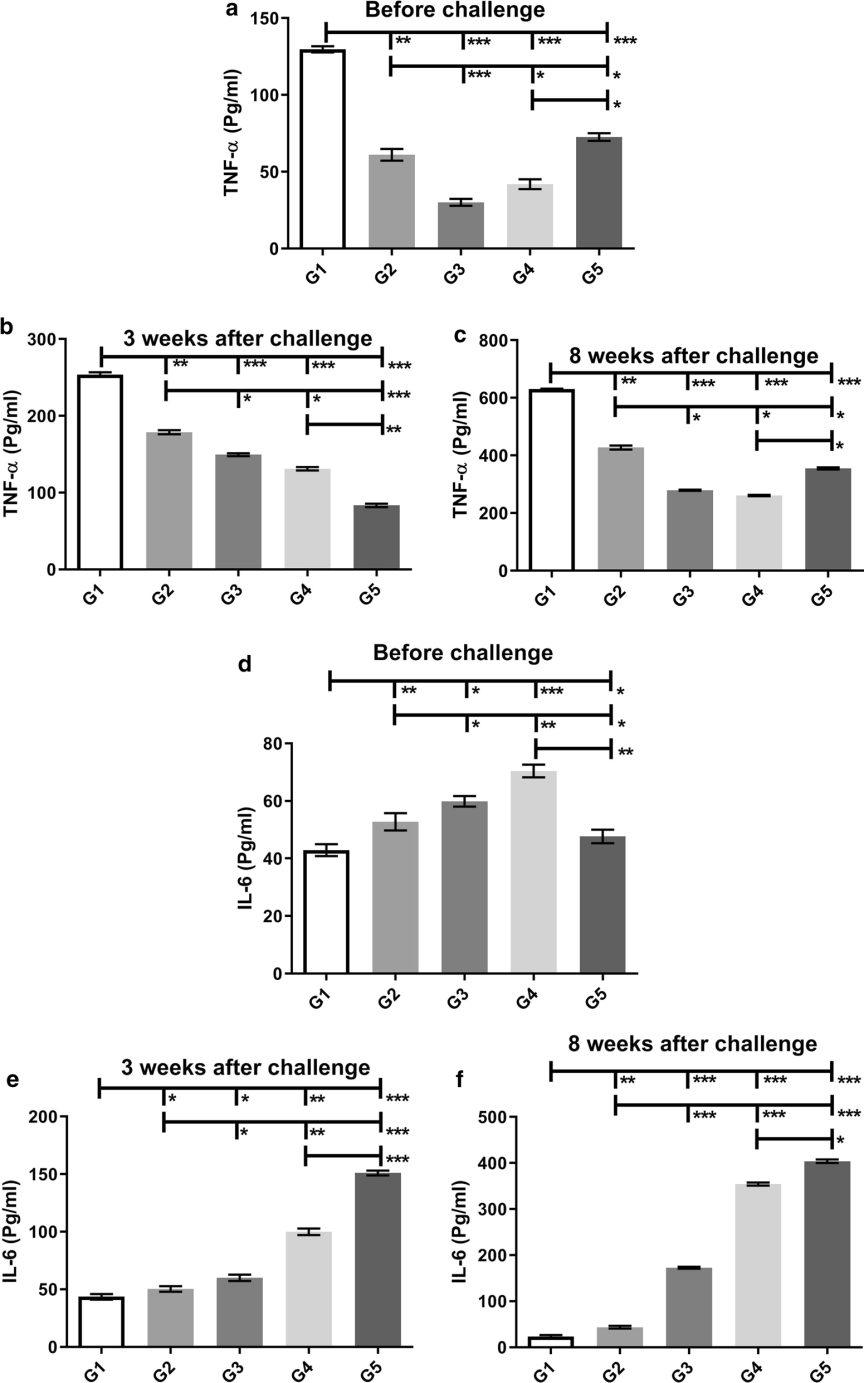
Analysis of the cellular immunity of vaccinated and control BALB/c mice. TNF-α (**a**–**c**), IL-6 (**d**–**f**) production from the splenocytes of mice after stimulation with *L. tarentolae*-LACK/KMP11/EGFP, *L. tarentolae/EGFP* (before challenge) and *L. major* F/T antigens (3 and 8 weeks after challenge) in different groups before and 3 and 8 weeks after challenge. All cytokines are presented as the mean ± SD in pg/ml in each bar. Statistical analysis of data was performed with Mann-Whitney U-test (*P < 0.05, **P < 0.001, ***P < 0.0001; ns, non-significant)

TNF-α had the highest level before and after the challenge in the G1 group compared to other groups (Mann-Whitney test: *U* = 0, *P *< 0.0001), indicating a shift in cellular immunity towards Th1 (Fig. 4a–c).

In addition, IL-6 was examined in all groups before and after the challenge. Before and three weeks after the challenge, the lowest level of IL-6 was seen in the G1 group compared to other groups (G2–G5) (Fig. 4d, e). On the 3rd week after the challenge, the G2 group also showed a decrease in the level of IL-6 production than before challenge compared to control groups, but this decrease was greater in the G1 group (Fig. 4e). Eight weeks following the challenge, the G1 group had the lowest level of IL-6 compared to other groups (G2–G5) (Mann-Whitney test: *U* = 0, *P *< 0.0001) indicating a shift in cellular immunity towards Th1 (Fig. 4f). At the same time, the G2 group also had shown the lowest level of IL-6 production compared to control groups after G1 group (Mann-Whitney test: *U* = 0, *P *< 0.0001) (Fig. 4f). On week 3 and 8 after the challenge, the G5 group had significantly the highest level of IL-6 in comparison to other groups (G1–G4) (Fig. 4e, f), indicating a shift in cellular immunity towards Th2.

**Fig. 5:**
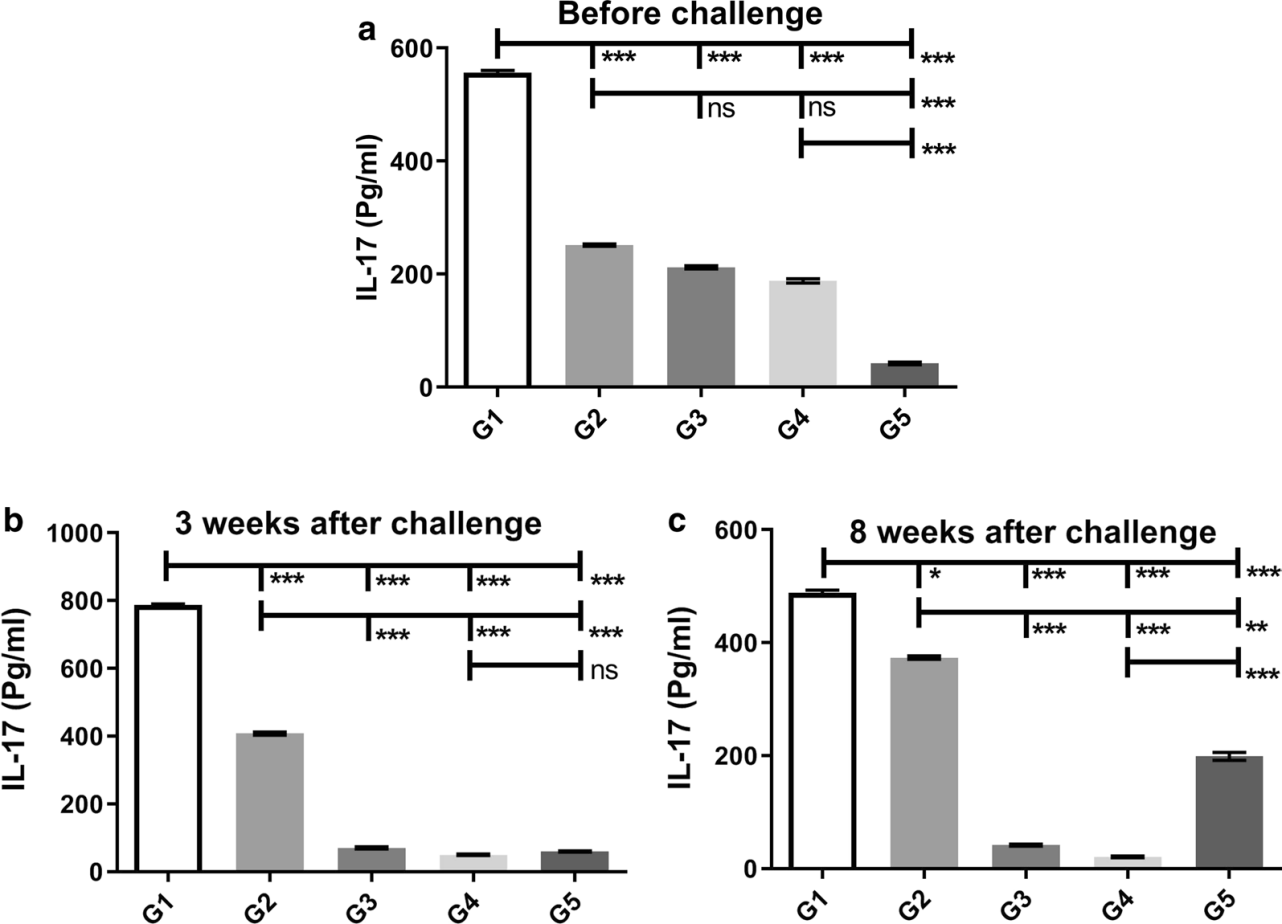
Analysis of the IL-17 cytokine production in the splenocytes of mice after stimulation with *L. tarentolae*-LACK/KMP11/EGFP, *L. tarentolae/EGFP* (before challenge) and *L. major* F/T antigens (three and eight weeks after challenge) before (**a**) and 3 (**b**) and 8 (**c**) weeks after challenge were evaluated by ELISA. All data are presented in the mean ± SD in pg/ml in each bar. Statistical analysis of data was performed with Mann-Whitney U-test (*P < 0.05, **P < 0.001, ***P < 0.0001; ns, non-significant)

The IL-17 cytokine is secreted from Th-17 induced by the CpG adjuvant, known as a protective cytokine, increasing cellular immunity to cutaneous leishmaniasis [22]. This cytokine was measured in all groups before and after the challenge. The G1 group showed the highest level of IL17 production compared to other groups (G2–G5) before and after the challenge (Mann-Whitney test: *U *= 0, *P *< 0.0001) (Fig. 5a, b). The G2 group followed by G3 and G4 groups had a higher level of IL-17 compared to the G5 group before the challenge, and eight weeks after the challenge, in response to stimulation with *L. major* antigen, G1, and G2 groups had the highest level of IL-17 compared to other groups (G3–G5) (Fig. 5c). On week 8 after the challenge, the G2 Group had shown an increase in the level of IL17 production compared to other control groups in response to stimulation with *L. major* antigen, but this increase was less than the G1 group (Mann-Whitney test: *U* = 0, *P *< 0.001) (Fig. 5c). The cytokine assays confirmed the humoral immunity assay results, showing a shift in cellular immunity towards Th1 in vaccinated mice.

### Measurement of footpad induration, ulceration and parasite load with real-time PCR

**Fig. 6:**
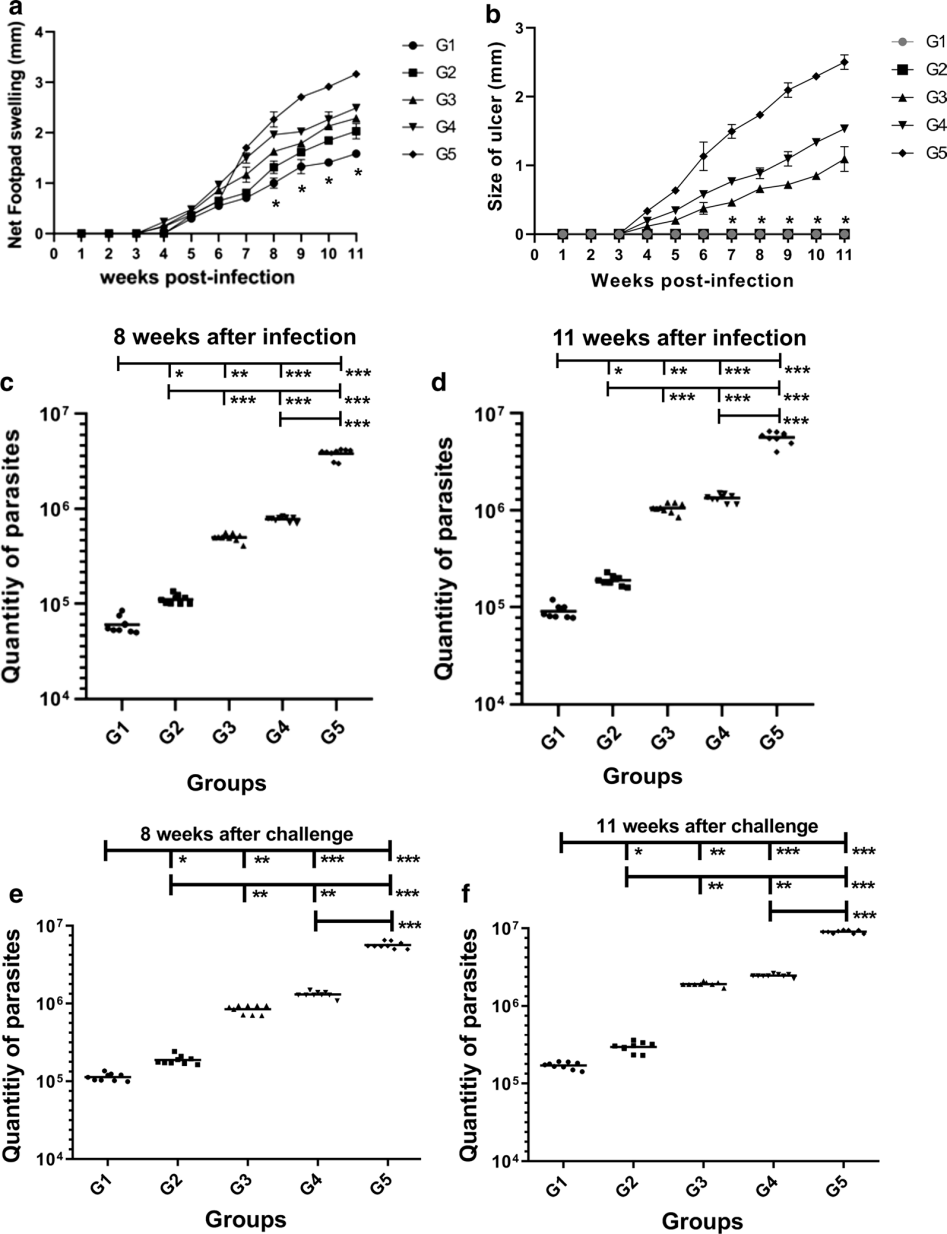
Measurement of footpad induration, ulceration and parasite load with Real-time PCR. Assessment of footpad induration (**a**) and ulceration (**b**) in vaccinated and control groups. The mean ± SD footpad induration and ulceration measurements calculated by vernier caliper in mm. BALB/c mice were immunized subcutaneously with *L. tarentolae*-LACK/KMP11/EGFP + CpG (G1, rLive vaccine); *L. tarentolae*-LACK/KMP11/EGFP + non-CpG (G2, rLive vaccine,); *L. tarentolae*-EGFP + CpG (G3, control); *L. tarentolae*-EGFP + non-CpG (G4, control); mice were injected with PBS (G5, control). One-way ANOVA with Tukeyʼs multiple comparison *post-hoc* tests were used for statistical analysis (the asterisk indicates the significant difference between values at the indicated time points). **c**–**f** Quantification of parasite burden in the popliteal lymph nodes (**c**, **d**) and in the footpad (**e**, **f**) in all groups at 8 and 11 weeks after the challenge was estimated by real-time PCR. All reactions were performed in triplicate. Statistical analysis of data was performed with Mann-Whitney U-test. (*P < 0.05, **P < 0.001, ***P < 0.0001; ns, non-significant)

The lowest footpad induration was observed in the G1 group vaccinated with *L. tarentolae*-LACK/KMP11/EGFP, compared to other groups from week 8 on, significantly differing with other groups. Moreover, on Weeks 8 and 11, the G1 group had the lowest induration among the groups (ANOVA: *F*_(4, 50)_ = 8.582, *P* < 0.05) (Fig. 6a). The highest induration was observed in group G5, a control group injected with PBS. In G2, G3 and G4 groups, no significant difference was observed eight weeks after the challenge (Fig. 6a). In groups 5, 4 and 3, at the site of wild parasite injection, a single ulcer was observed at 11 weeks, and lesions of about 2.5 mm (intermediate-sized), 1.5 mm, and 1 mm were observed in groups 5, 4 and 3, respectively. In groups G1 and G2, non-ulcerated induration was observed (Fig. 6b). Subsequently, to examine the effectiveness of the vaccine, the parasite load in the popliteal lymph node (Fig. 6c, d) and in the footpad (Fig. 6e, f) at weeks 8 and 11 after the injection of *L. major* were estimated in vaccinated and control mice with the aid of RV1 and RV2 primers. The lowest parasite load belonged to G1 and G2 groups compared to G3, G4 groups, and G5 as a control group showing a significant difference (Mann-Whitney test: *U* = 0, *P *< 0.0001) between the G1 group vaccinated with *L. tarentolae-LACK/KMP11/EGFP* with CpG adjuvant showed the lowest parasite load compared to other groups. This difference in parasite load showed the highest level of immunogenicity of the vaccine in this group compared to the G2 group at weeks 8 and 11 (Fig. 6b–e). This could be due to the effect of the adjuvant in this group and a shift in the immune profile towards Th1. Also, the measurement of footpad induration and parasite load with real-time PCR in the G1 group confirmed the cytokine and humoral immunity assay findings which showed post-vaccination immune profile shift to Th1 and decreased induration and parasitic burden as well.

## Discussion

Despite extensive efforts to prepare an appropriate vaccine for the control and treatment of leishmaniasis, this disease is still threatening the lives of human beings. Antimony compounds have been used for the treatment of this disease for years, and resistance against pentavalent antimony (SbV) has significantly been increased [23]. Achievement of an effective vaccine is essential for this disease. In the present study, the sequences of KMP11 and LACK *L. major* antigens in the form of molecular constructs were fabricated inside the pLEXSY-neo 2.1 vector (Jena Bioscience, Germany) and cloned in *E. coli* strain Top10, and after linearization with *Swa*I enzyme, they were stably transfected inside the *L. tarentolae* genome. After the selection of recombinant strains using neomycin antibiotics, they were confirmed by PCR and Western blot, and the recombinant parasite was produced and injected to the mice together with the CpG ODN adjuvant. This recombinant parasite was evaluated as a recombinant vaccine for the control and treatment of leishmaniasis in BALB/c mice, and immune profiles were then examined. So far, these two antigens had not been evaluated simultaneously using a live recombinant vector and/or *L. tarentolae*. In addition, in this study, the induction of immune cells using *L. tarentolae* was performed using immunogenic antigens of *L. major* synthesized with a eukaryote system and glycosylation, which resulted in better immunogenicity.

CpG ODN is a TLR9 agonist and can mediate natural and inherent immunity when used as an adjuvant [24]. Previous studies have examined the effect of CpG ODN as an adjuvant on acquired immunity and reduction of the intensity of infection in parasitic, viral, and bacterial diseases [25, 26]. In the present study, a recombinant *L. tarentolae*-LACK/KMP11/EGFP vaccine with CpG adjuvant increased IFN-γ, IL-17, and TNF-α cytokines.

In this study, the lowest footpad induration was observed in G1 vaccinated with *L. tarentolae*-LACK/KMP11/EGFP and CpG adjuvant compared to other groups from week 8 on, showing significant differences from other groups. Since the measurement of footpad induration alone is not a precise evaluation of the level of immunogenicity, the parasite load was also evaluated in the vaccinated and control groups using real-time PCR, which has higher precision than microscopic observation [27]. The high sensitivity of this method is due to a large number of copies of this gene [27]. One research has demonstrated that nuclear DNA and kinetoplast DNA are rapidly decomposed after the death of the parasite, and the results of the PCR test only show the DNA derived from live parasites [27]. With the use of RV1 and RV2 primers in vaccinated and control mice, the parasite load in the popliteal lymph node and in the footpad were estimated, with G1 group mice vaccinated with *L. tarentolae*-LACK/KMP11/EGFP and CpG adjuvant showing a significantly lower level of parasite load in comparison with the other groups. Moreover, IFN-γ had the highest level before and after injection of *L. major* in the G1 group compared with the other groups (*P <* 0.05), indicating a shift in cellular immunity towards Th1 and protection. IL-5 is a cytokine-dependent on Th2, with the lowest level in the G1 group before and after injection of *L. major* compared to other groups (*P <* 0.05). The IFN-γ/IL-5 ratio was also calculated after the injection of *L. major*, with the highest level in G1 at 3 and 8 weeks after the injection compared to other challenge groups and the control groups, showing a shift towards Th1. TNF-α had the highest level before and after the challenge in the G1 group among the groups (*P <* 0.05), demonstrating a shift in cellular immunity towards Th1 and an absence of lesion formation. IL-6 had the lowest level at 8 weeks after the challenge in the G1 group compared to the other groups (G2–G5) (*P <* 0.05), indicating a shift in cellular immunity towards Th1. On weeks 3 and 8 after the challenge the G5 group had significantly the highest levels of IL-5 and IL-6 in comparison to other groups, indicating a shift in cellular immunity towards Th2.

In a study by Katebi et al. [25], *L. tarentolae* was used as a recombinant live vaccine with CpG adjuvant to produce sand fly salivary antigen. In this study, the difference in the level of cytokines IL-5 and IL-6 in the group vaccinated with this adjuvant was observed [25]. In our study, this difference also was observed in the group G1 mice vaccinated with *L. tarentolae*-LACK/KMP11/EGFP and CpG adjuvant and the group G2 mice vaccinated with *L. tarentolae*-LACK/KMP11/EGFP plus non-CpG.

In this study, because of our limitations, only IL-5 and IL-6 were measured by ELISA to evaluate the Th2 polarization even though it would be better to evaluate IL-4 and IL-10. Therefore, more detailed cytokine evaluation is needed to more precisely characterize Th1/Th2 polarization induced by the vaccine.

CpG increases Th17 and the production of IL-17, thereby increasing pro-inflammatory and inflammatory cytokines, recalling other leukocytes to the vaccination site, and directing immune responses towards Th1 [22]. In this study, in the G1 group, CpG was used as an adjuvant along with the recombinant live vaccine, which had shown the highest levels of IL-17 before and after injection of *L. major* in comparison with the other groups (*P <* 0.05).

The cellular immunity assay confirmed the humoral immunity assay results. The highest level of IgG2a/IgG1 ratio was observed in the G1 group which was vaccinated with the recombinant *L. tarentolae*-LACK/KMP11/EGFP along with the CpG adjuvant (*P <* 0.05) both before and six weeks after the injection of *L. major* parasite. This indicates a shift in immunity towards Th1, also confirmed by cellular immunity findings.

In the study conducted by Flynn et al. [28], the effect of CpG ODN on the treatment of cutaneous leishmaniasis lesions was examined in macaques. In their study, the CpG ODN was effective when used before and after the injection of the wild parasite. The macaques receiving CpG ODN before injection had smaller lesions compared to those receiving it after the injection, and their lesions were repaired more rapidly [28]. The use of CpG ODN in addition to leishmanization in mice, as compared to the use of leishmanization alone, increases immunogenicity and reduces lesion formation, thus exerting a better effect [25, 29]. In our study, the use of this adjuvant along with the recombinant live vaccine increased immunogenicity, and decreased induration and also no ulcer was observed in vaccinated groups.

Despite extensive efforts to control leishmaniasis, no effective vaccine has yet been discovered for humans, and this disease is still threatening human lives [30]. The application of live attenuated vaccines always runs the risk of reversibility of the vaccine to the wild state, increasing the sources of infection in the human population. *L. tarentolae* is not attenuated but is non-pathogenic to humans. The risk of reversion by *L. tarentolae* to a pathogenic strain is essentially zero because the species is inherently non-pathogenic to humans, making it safer than a live attenuated vaccine.

In this study, the use of *L. tarentolae* as a live factory for the live production of these antigens led to superior immunogenicity and increased cellular immunological memory; thus, the vaccinated mice had no ulcerated lesions.

In this study, the live recombinant vaccine with CpG adjuvant showed the reduction of parasitic load and footpad induration against needle injection of *L. major* in mice. A study by Peter et al. [31] has shown that the autoclaved *L. major* antigen (ALM) coupled with CpG vaccine showed good results against needle injection of *L. major* in BALB/c mice, but when exposed to natural vector transmission by infected sand fly bites, it failed and showed low efficacy [31]. In the case of the recombinant live vaccine used in this study, it is best to evaluate its efficacy in vaccinated mice exposed to natural vector transmission by infected sand fly bites before testing the vaccine in humans.

In another study, the sand fly salivary antigen (PpSP15 (with CPA and CPB antigens produced as a recombinant live vaccine in *L. tarentolae* were evaluated against cutaneous leishmaniasis in BALB/c mice. The use of salivary gland antigen showed increased efficacy of the vaccine used [20]. In the live recombinant vaccine applied in the present study, the structure of the construct can also be modified so that the salivary antigen can be produced in a live and recombinant manner after the vaccine is injected into the mouse body.

In this study, we evaluated the efficacy of recombinant live vaccines against wet cutaneous leishmaniasis, which is one of the major health problems in Iran. However, it can also be evaluated on urban-type leishmaniasis. As ACL leishmaniasis does not have a suitable laboratory animal for vaccine evaluation, it is advisable to be tested by clinical trials in volunteers after further evaluation.

This method of preparation of recombinant live vaccine can be used to evaluate other species of *Leishmania*. Although *Leishmania* antigens are widely homologous in different species, it is better to use specific antigens of each strain for superior vaccine efficacy.

The KMP11 antigen is present in membrane structure, cell surface, intracellular vesicles, and flagellar pocket and it is expressed in both the amastigote and the promastigote stages. It is more expressed at the cell surface in amastigotes and metacyclic promastigotes, indicating the importance of this antigen in relation to the mammalian host. It has strong antigenicity to stimulate mouse and human T cells is capable of stimulating both innate and acquired immune systems and has low homology with human proteins making it a suitable candidate for the vaccine use [32]. In the study of Ramirez et al., *Toxoplasma gondii* ts-4 engineered mutants were used to express KMP11. This recombinant live vaccine was evaluated for the treatment of rural type leishmaniasis. In this study, in vaccinated mice, increased IFN-γ production and decreased wound induration were observed in mice [33]. These results are consistent with our study, where increased IFN-γ and decreased wound induration were observed in the vaccinated groups. We used *L. tarentolae* to produce recombinant live vaccines that are easier and safer to produce in the laboratory model. It is also cultivable *in vitro* and does not require a laboratory animal for culture.

In the study of Hugentobler et al. [34] used *Lactococcus lactis* to develop a recombinant live vaccine. They examined the LACK *L. major* antigen with active single-chain mouse IL-12 as a recombinant live vaccine in BALB / c mice against rural leishmaniasis. In this study, the immune profile shifted to Th1, and delayed induration was observed in vaccinated mice. The present study employed the eukaryote host (*L. tarentolae*) to construct a recombinant live vaccine capable of producing eukaryotic antigens rather than prokaryotic ones.

Two *L. major* immunogen antigens were used instead of one antigen with the CpG adjuvant, a TLR9 antagonist that increased IL-17, induced a shift of immune profile to Th1 and decreased lesion induration and parasitic load in vaccinated groups.

## Conclusions

To the best of our knowledge, this was the first time that the LACK and KMP11 antigens were prepared in the form of a single recombinant live vaccine and examined along with the CpG adjuvant. Based on the results of this study, the recombinant live *L. tarentolae*-LACK/KMP11/EGFP vaccine with the CpG adjuvant used against rural cutaneous leishmaniasis showed good effects on BALB/c mice. To evaluate the long-term effects of this vaccine due to the short lifespan of BALB/c mice, this vaccine can be subsequently tested in volunteers using a clinical trial.

## Data Availability

Data supporting the conclusions of this article are included within the article.

## Abbreviations

CL: cutaneous leishmaniasis
LACK: *Leishmania* homologue of the receptor for the activated C kinase
KMP11: Kinetoplastida membrane protein-11
IFN-γ: interferon γ
IL-5: interleukin-5
IL-17: interleukin-17
IL-6: interleukin-6
TNF-α: tumor necrosis factor alpha
